# In this month’s Bulletin

**DOI:** 10.2471/BLT.24.001224

**Published:** 2024-12-01

This month’s theme issue on sexual health is introduced in an editorial by Manjulaa Narasimhan and Pascale Allotey (842). In related editorials, Sagri Singh et al. (843) advocate for improved sexual health and well-being and Nicaise Ndembi et al. (844) explain how mpox poses a new threat. Etienne V Langlois et al. (845) present a critical pathway for the reduction of maternal newborn and child mortality.

Gary Humphrey (848–849) reports on efforts to communicate effectively about mpox transmission while avoiding stigmatization. Haven Ley talks to Humphreys (850–851) about Pivotal Ventures, established to further social progress and address women’s sexual health and reproductive rights.

## Australia, Bangladesh, Botswana, Brazil, Canada, Colombia, Ghana, Guinea, Indonesia, Italy, Kenya, Malaysia, Myanmar, Nigeria, Pakistan, Thailand, Uganda, Uruguay

### WHO’s Sexual Health Assessment of Practices and Experiences

Erin C Hunter et al. (861–872) refine a survey instrument.

## Australia, Brazil, Canada, China, Taiwan, Denmark, India, Islamic Republic of Iran, Italy, Poland, Portugal, Republic of Korea, Spain, Tunisia, Türkiye, United Kingdom of Great Britain and Northern Ireland, United States of America 

### Associations between sexual health and well-being

Priscila Vasconcelos et al. (873–887) review the available evidence.

## WHO African region 

### Managing HIV 

Sharana Mahomed et al. (913–915) describe a women’s life course approach.

## United Republic of Tanzania

### Improving HIV prophylaxis for female sex workers

Christopher H Mbotwa et al. (852–860) test an mHealth intervention.

## Global

### Sexual exploitation in humanitarian contexts

Jasmine-Kim Westendorf et al. (888–894) outline gaps in research on prevention.

### Sustainable development goals

Onikepe O Owolabi et al. (895–903) summarize progress related to sexual health.

### Menstruation and sexual health

Carmen H Logie et al. (904–906) show links to well-being and justice.

### Measuring sexual empowerment

Anna E Kågesten et al. (907–909) propose improved metrics.

### Ensuring respectful sexual health services

Rosemary Morgan et al. (910–912) detail ways to address harmful gender norms.

### Sexual health in later life

Linda McAuliffe and Deirdre Fetherstonhaugh (916–918) consider the needs of older adults.

### Understanding endometriosis 

Nilufer Rahmioglu and Krina T Zondervan (919–921) define health disparities and research gaps.

### A global picture of perimenopause

Yvette Pyne et al. (922–924) note persistent inequalities in diagnosis and management.

